# Selenium-Enriched Pollen Grains of *Olea europaea* L.: Ca^2+^ Signaling and Germination Under Oxidative Stress

**DOI:** 10.3389/fpls.2019.01611

**Published:** 2019-12-11

**Authors:** Alberto Marco Del Pino, Luca Regni, Roberto D’Amato, Emma Tedeschini, Daniela Businelli, Primo Proietti, Carlo Alberto Palmerini

**Affiliations:** Department of Agricultural, Food and Environmental Sciences, Università degli Studi di Perugia, Perugia, Italy

**Keywords:** olive, se-fertilization, selenium, se-methionine, cytosolic Ca^2+^, pollen germination

## Abstract

Selenium (Se) shows antioxidant properties that can be exploited in plants to combat abiotic stresses caused by reactive oxygen species produced in excess (ROS). Here, we show that the Se-fertilization of olive trees with sodium selenate effectively protects the pollen from oxidative stress. Pollen isolated from plants treated with Se or from untreated controls was incubated *in vitro* with H_2_O_2_ to produce an oxidative challenge. Given the impact of ROS on Ca^2+^ homeostasis and Ca^2+^-dependent signaling, cytosolic Ca^2+^ was measured to monitor cellular perturbations. We found that H_2_O_2_ interrupted Ca^2+^ homeostasis only in untreated pollen, while in samples treated *in vitro* with sodium selenate or selenium methionine, Ca^2+^ homeostasis was preserved. Furthermore, germination rates were considerably better maintained in Se-fertilized pollen compared to non-fertilized pollen (30% vs. 15%, respectively) after exposure to 1 mM H_2_O_2_. The same was observed with pollen treated *in vitro* with Se-methionine, which is the organic form of Se, in which part of the fertigated sodium selenate is converted in the plant. Combined, our results show a close correlation between ROS, Ca^2+^ homeostasis, and pollen fertility and provide clear evidence that Se-fertilization is a potential approach to preserve or improve agricultural productivity.

## Highlights

1. Cytosolic Ca2+ of Se-enriched pollen is not deregulated by oxidative stress.
2. Toxic effects of H_2_O_2_ on germination are strongly mitigated in Se-enriched pollen.
3. Se foliar fertilization increases the concentration of organic selenium in pollen.

## Introduction

Selenium (Se), a microelement, has been used for many years in disease prevention (Boosalis, 2008; Rayman, 2008; Rees et al., 2013; D’Amato et al., 2014). However, as a food additive, it is used with caution because of its high toxicity. Although so far, it has not been confirmed as an essential micronutrient for trees, there is increasing evidence that it plays a role in abiotic stress, causing an overproduction of reactive oxygen species (ROS) (Lyons et al., 2009; Prins et al., 2011; Proietti et al., 2013; Tedeschini et al., 2015; D’Amato et al., 2017). Although ROS are normally produced by cells at low concentrations and participate in membrane signals and cell events, such as sexual *plant* reproduction (recognition between pollen and stigma), their accumulation leads to oxidative stress due to the loss of cell scavenging capacity (Hancock et al., 2001; Laloi et al., 2004; Kwak et al., 2006).

Higher plants have a specialized sexual reproduction system with the ability to produce large amounts of male sperm, transported over great distances *via* wind or insects, which has facilitated the colonization of various habitats and adaptation to dry environments (Michard et al., 2009).

In angiosperms, after landing on the stigma, dehydrated pollen quickly hydrates and begins to germinate. The germination of the pollen grain and the correct lengthening of the pollen tube are essential processes of the sexual reproduction of plants (Lazzaro et al., 2005; Wu et al., 2011).

Numerous signals help to guide the male spermatozoa towards the haploid egg cell (the female gametophyte), and signal-response coupling is of fundamental importance in all cells and is mostly mediated by ions (Kwak et al., 2006; Michard et al., 2009).

The second cytosolic Ca^2+^ messenger has been one of the main study objectives to highlight its role in plant cells (Miller et al., 1992; Sanders et al., 2002; Hetherington and Brownlee, 2004; Dodd et al., 2010; Steinhorst and Kudla, 2013). In the plasma membrane of the plant cell, there are several channels that mediate the entry of Ca^2+^, of which, despite being permeable to the ion, none is selective to Ca^2+^. Through these channels, the ion enters the cell to activate vertical growth (Michard et al., 2009). Also in the pollination process, the regulation of the growth signal leading to the formation of the pollen tube is Ca^2+^-dependent (Campanoni and Blatt, 2006; Cheung and Wu, 2008; Michard et al., 2009). When ROS are produced at high concentrations, as under biotic stress, they become toxic by altering the molecular signals of the cell, including cytosolic Ca^2+^. The accumulation of ROS in oxidative stress determines the infertility of pollen, with negative repercussions on fertilization and, consequently, on agricultural production (Carafoli, 1987; Görlach et al., 2015).

A rich literature describes the correlation between Ca^2+^ and ROS, but few studies have investigated the effects of selenium on cytosolic Ca^2+^ and pollen germination (Steinhorst and Kudla, 2013; Görlach et al., 2015).

In this work, in an attempt to fill this gap, the role of selenium in the two biological events was investigated. The study was conducted by determining the cytosolic Ca^2+^ and germination in pollen grains from plants Se-fertilized and from control plants under the conditions of oxidative stress.

The ROS behave like agonists, stimulating the mobilization of the ion from the "internal stores" and activating the entry of Ca^2+^ from the extracellular medium (Ca^2+^-entry) (Carafoli, 1987; Yan et al., 2006; Clapham, 2007; Brini et al., 2013; Görlach et al., 2015; Orrenius et al., 2015)

## Materials and Methods

### Reagents

The substances FURA 2-AM (FURA-2-pentakis (acetoxymethyl) ester, Triton X-100 (t-octylphenoxypolyethoxyethanol), EGTA (ethylene glycol-bis (β-aminoethyl ether)-N,N,N’,N’-tetracetic acid), sodium selenite (Na_2_SeO_3_), sodium selenate (Na_2_SeO_4_), selenomethionine (SeMet), selenocysteine (SeCys), Se-(methyl)selenocysteine (Se SeMeSeCys), and protease (Protease TypeXIV), as well as olive pollen, were purchased from the Sigma-Aldrich corporation (St. Louis, MO, USA), along with nitric acid (HNO_3_ 65% RPE), dimethyl sulfoxide (DMSO), hydrogen peroxide (H_2_O_2_ 40% wv RE pure), and other reagents (reagent grade).

### Plant Material, Growing Conditions, and Pollen Collection

The study was carried out in 2018 on *O. europaea* L. trees, cultivar Leccino, grown in a 30-year-old orchard near Perugia (Central Italy, 42°57’39.2"N, 12°25’02.5"E). The soil is clay loam, and the trees were trained to the vase system (with a 1-m-high trunk and three to four main branches) at a planting distance of 5 × 6 m. The area has a semi-continental climate; the average temperature difference between the coldest (January) and hottest (July) months is 19–20°C (with an average diurnal thermal range of 10–11°C and an average annual air temperature of 13–14°C). The maximum and minimum temperatures are 36 and –7°C, respectively. Average annual precipitation is about 800 mm, distributed mostly in autumn, winter, and spring (Proietti et al., 2014). The olive grove is considered to be representative of many olive groves in central Italy.

In 2017 (April 15^th^), 10 trees with uniform size and vegetative characteristics were sprayed with a solution containing 100 mg L^-1^ of Se, following the procedure described in D’Amato et al. (2018). This solution was obtained by dissolving sodium selenate (SeO_4_^2-^) in water. For each treatment, 0.5% of the Albamilagro wetting agent (Albamilagro International S.p.A., Parabiago, MI, Italy) was added, and each plant was treated with 10 L of Se solution. At the base of the tree, a filter paper, impermeable at the side in contact with the soil, was placed to prevent the solution from dripping onto the soil. The paper was weighed before and after spraying to calculate the amount of solution that was absorbed by the plant, which was 8.0 ± 1.2 L. Twenty randomly selected "control" trees were sprayed with the same technique, but with a water solution containing only the wetting agent (mock treatment). All trees reached the first stage of flowering between June 4 and 6, 2018. The phenology assessment of olive initial flowering was established when the pollen was freely released by shaking the anthers of different branches, located at different heights on the tree and with different exposures (see Tedeschini et al., 2015).

At the beginning of the flowering phase, three branches for each tree (treated and control) were bagged using white double-layer paper bags (0.65 × 0.35 m) for pollen collection. The branches had 70–80 inflorescences each. At the end of the flowering phase, the bags were removed and the pollen was filtered through a cell strainer (40 µm).

### Determination of Total Selenium in Olive Pollen

Measurements of total selenium contents in olive pollen were performed using defrosted and dry samples, respectively. Pollen samples (0.5 g sample^-1^) were microwave-digested (ETHOS one high-performance microwave digestion system; Milestone Inc., Sorisole, Bergamo, Italy) with 8 ml of ultrapure concentrated nitric acid (65% w/w) and 2 ml of hydrogen peroxide (30% w/w). The heating program for the digestion procedure was 30 min at 1,000 W and 200°C. After cooling down, the digests were diluted with water up to 20 ml and passed through 0.45-μm filters. The analysis was conducted using a graphite furnace atomic absorption spectrophotometer, Shimadzu AA-6800 apparatus (GF-AAS; GFA-EX7, Shimadzu Corp., Tokyo, Japan) with deuterium lamp background correction and a matrix modifier [Pd(NO_3_)_2_, 0.5 mol L^‑1^ in HNO_3_]. All analyses were carried out in triplicate.

### Se Speciation With HPLC ICPMS

Fresh shoot material (0.25 g) was mixed with 10 ml of the 2.0 mg ml^‑1^ protease solution. Samples were sonicated with an ultrasound probe for 2 min and stirred in a water bath at 37°C for 4 h. Subsequently, they were cooled at room temperature and centrifuged for 10 min at 9,000 rpm. The supernatant was filtered through 0.22-μm Millex GV filters (Millipore Corporation, Billerica, MA). The standards solutions (1, 5, 10, and 20 μg L^‑1^) for inorganic (i.e., selenite, SeO_3_^‑2^ and selenate, SeO_4_^‑2^) and organic [i.e., selenocystine (SeCys2), Se-(methyl) selenocysteine (SeMeSeCys), selenomethionine (SeMet)] Se forms were prepared with ultrapure (18.2 MΩ cm) water. Speciation of Se was performed by HPLC (Agilent 1100, Agilent Technologies, USA) using an anion exchange column (Hamilton, PRP-X100, 250 × 4.6 mm^2^, 5 μm particle size). The mobile phase consisted of ammonium acetate with gradient elution. The analytical method and instrumental conditions have been described previously (Proietti et al., 2018).

### Measurement of Cytosolic Ca^2+^

Intracellular calcium levels were determined spectrofluorometrically using a FURA-2AM probe (Koubouris et al., 2009). For this, 100 mg of olive pollen was suspended in 10 ml PBS and hydrated for 3 days.

Hydrated pollens were harvested by centrifugation at 1,000 g x 4 min and then resuspended in 2 ml Ca^2+^-free HBSS buffer (120 mM NaCl, 5.0 mM KCl, MgCl_2_ 1mM, 5 mM glucose, 25 mM Hepes, pH 7.4). Pollen suspensions were incubated in the dark with FURA-2 (2 µl of a 2 mM solution in DMSO) for 120 min, after which the samples were centrifuged at 1,000 g x 4 min. Pollens were then harvested and suspended in ~10 ml of Ca^2+^-free HBSS containing 0.1 mM EGTA, which was included to rule out or, at least, minimize a potential background due to contaminating ions (to obtain a suspension of 1 x 10^6^ of pollen granules hydrated per ml).

Fluorescence was measured in a Perkin-Elmer LS 50 B spectrofluorometer (ex. 340 and 380 nm, em. 510 nm), set with a 10-nm and a 7.5-nm slit width in the excitation and emission windows, respectively. Fluorometric readings were normally taken after 300–350 s. When required, samples of pollen, CaCl_2_, H_2_O_2_, and Na_2_SeO_4_ were added for specific purposes, as described in the *Results* section.

Cytosolic calcium concentrations [Ca^2+^]_c_ were calculated as shown by Grynkiewicz (Koubouris et al., 2009).

### Pollen Germination

The olive pollen grains used in the experimentation were derived from treated trees in the field (Se-enriched) and from control trees.

Freshly collected pollen samples were rehydrated by incubation in a humid chamber at room temperature for 30 min (Fontanella et al., 2017) and subsequently transferred to a culture plate [6-well culture plate (1.0 mg of pollen per plate)] containing 3 ml of an agar-solidified growing medium: agar 1%, sucrose 10%, boric acid (H_3_BO_3_) 100 ppm and calcium chloride (CaCl_2_) 1 mM, at pH 5.5 (Grynkiewicz et al., 1985). Subsequently, with the aid of a brush, a uniform distribution was obtained on the surface of the medium. Pollen grains then were incubated for 24–48 h in a growth chamber at 25°C.

The numbers of germinated and non-germinated pollen grains were determined with the aid of a microscope with a 10x objective lens. Germination rates were determined using two replicates of 100 grains. Grains were considered germinated if the size of the pollen tube was greater than the diameter of the grain (Rejón et al., 2012). Experiments were conducted in a completely randomized design with four replications.

### Statistical Analysis

Statistical tests were performed using the Graph Pad Prism 6.03 software for Windows (La Jolla, CA). Tests for variance assumptions were conducted (homogeneity of variance by Levene’s test, normal distribution by the D’Agostino-Pearson omnibus normality test). The results are expressed as mean values ± standard error of the mean (SEM). Significance of differences was analyzed by Fisher’s least significant differences test, after analysis of variance (**Supplementary Tables 1**–**6**).

## Results

### Determination of Total Se and Speciation in Pollen Grains

Pollen grains, collected in June from Se-enriched and control trees, were analyzed for HPLC ICPMS. Based on the results, the SeO_4_^2-^ distributed with the treatment was absorbed by olive trees and organicated for ≈ 50% as Se-methionine (Se-Met) in the pollen grains (**Table 1**).

**Table 1:** Speciation of selenium in olive pollen grains.

ppb	SeCys	MetSeCys	SeMet	Se(IV)	Se (VI)	Se tot
Control	<0.1	<0.1	115 ± 11	30 ± 3	172 ± 12	321 ± 15
Se-enriched	15 ± 2	15 ± 3	1,489 ± 35	11 ± 2	1,586 ± 33	3,837 ± 52

Results are expressed as means ± SEM from three independent measurements.

### Oxidative Stress Induced “*In Vitro*” With H_2_O_2_ in Control and Se-Enriched Olive Pollen Grains

The marking of control olive pollen grains with the FURA 2-AM fluorescent probe allowed the determination of the variations in the time of the pollen cytosolic calcium [Ca^2+^]_cp_ under different experimental conditions.

The levels of [Ca^2+^]_cp_ were ≈15–30 nM in baseline conditions and increased in the presence of H_2_O_2_ in the incubation medium in a dose-dependent manner. The experiment was conducted in two phases, initially in the absence of Ca^2+^ in the incubation medium and subsequently with the addition of CaCl_2_ (1 mM) after 250 s.

**Figure 1:**
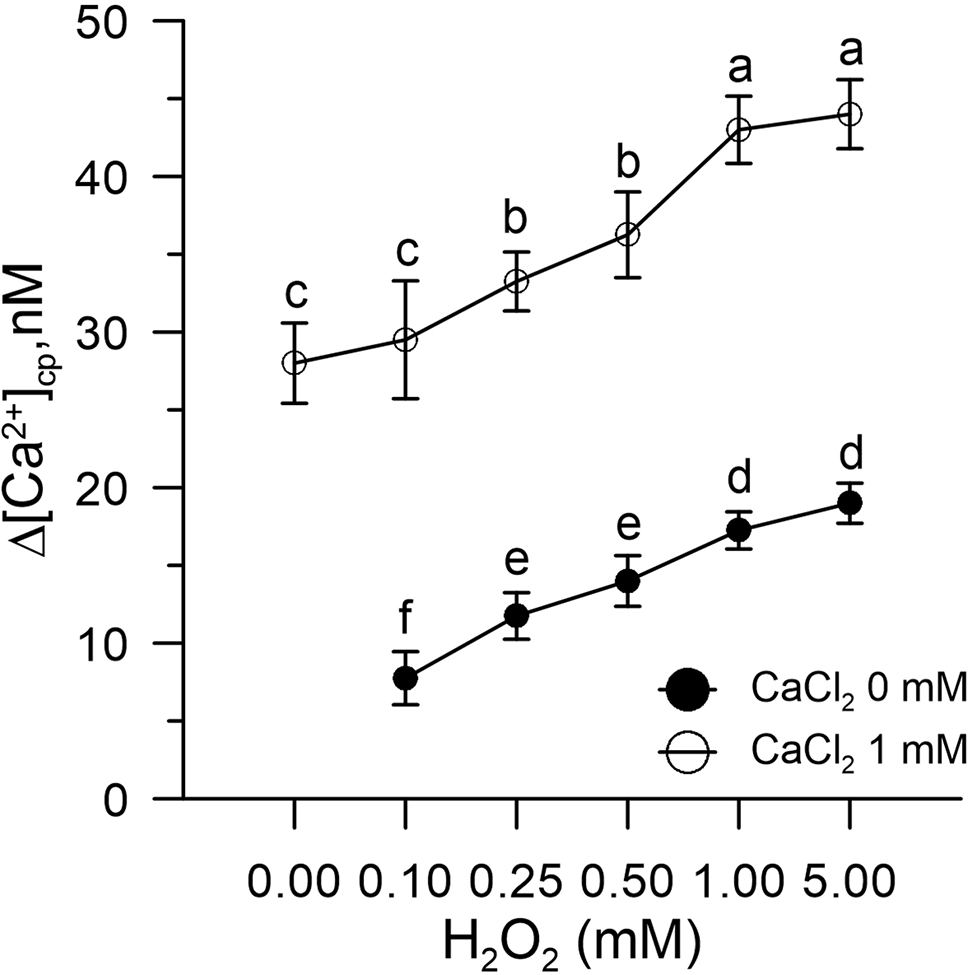
Effect of H_2_O_2_ (0.1–5.0 mM) on [Ca^2+^]_cp_ in control pollen grains in the presence (○) and absence (•) of CaCl_2_ in the incubation medium. Results are expressed as Δ[Ca2+]cp (nM) to reflect signal changes detected between the start and the end of the fluorometric measurements and represent means ± SEM from four independent tests. Statistically significant differences between ○ and • are indicated by different letters, whereas identical letters highlight non-significant trends.

We found that H_2_O_2_-induced oxidative stress caused a significant alteration of Ca^2+^ homeostasis, evidenced by an increase in cytosolic Ca^2+^ and a marked enhanced Ca^2+^ entry when exogenous 1 mM CaCl_2_ was added to the pollen suspension (**Figure 1**).

### Effects of Na-Selenate (SeO_4_^2-^) and Se-Methionine (Se-Met) on [Ca^2+^]_cp_ During Induced Oxidative Stress

Control pollen grains marked with FURA-2AM and pre-incubated *in vitro* with SeO_4_^2-^ or Se-Met (3.4 µM) did not show changes in [Ca^2+^]_cp_ levels after the addition of H_2_O_2_ (0–5 mM).

**Figure 2:**
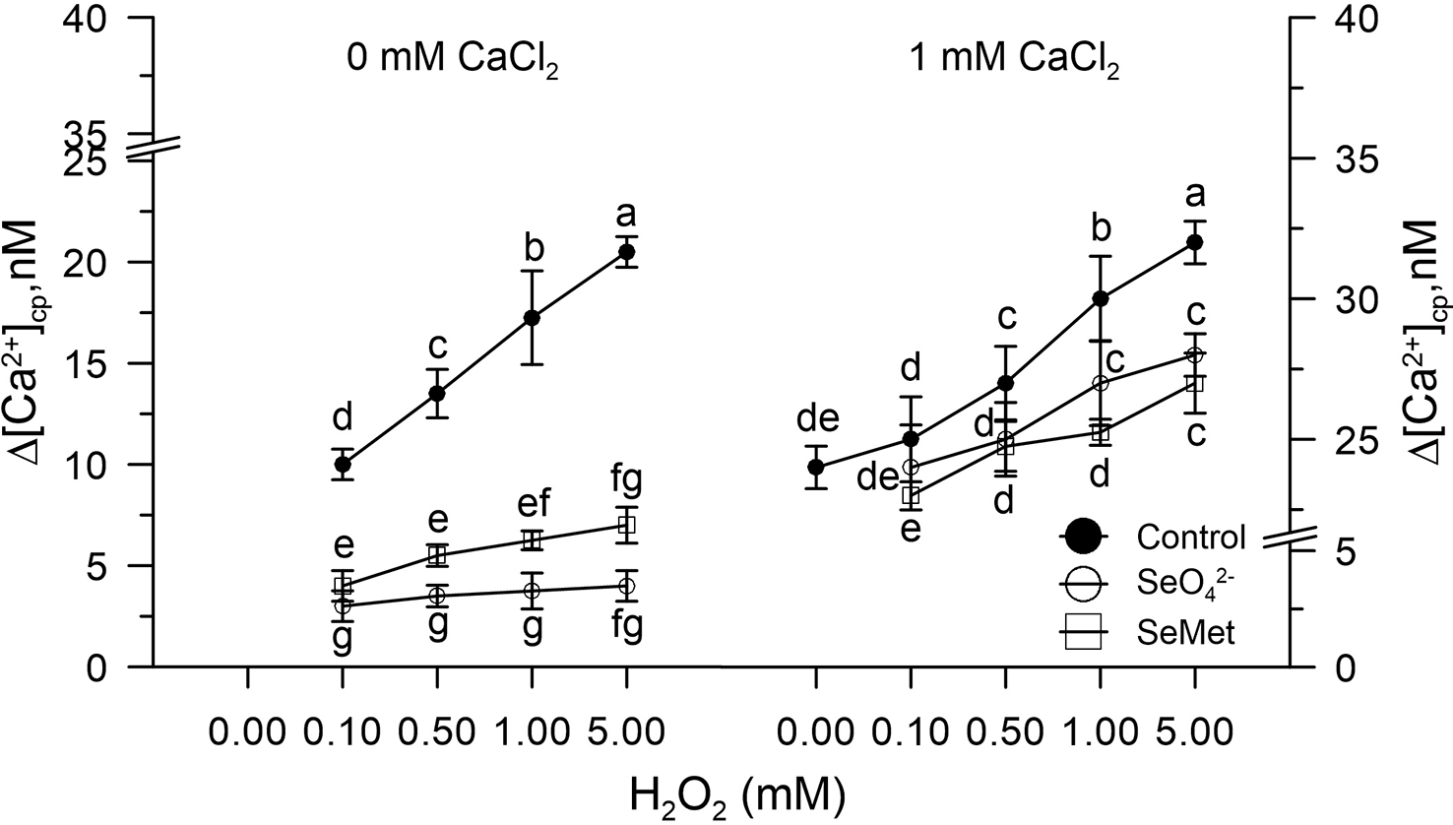
Effects of sodium selenate (SeO_4_^2-^ 3.4 µM) or selenium methionine (SeMet 3.4 µM) on [Ca^2+^]_cp_ in pollen grains subjected to oxidative stress induced with H_2_O_2_ (0–5 mM), in the presence and absence of CaCl_2_ in the incubation medium. Changes in cytosolic [Ca^2+^]_cp_ were assessed in control pollen grains exposed to SeO_4_^2-^ or SeMet employed at a dose of 3.4 µM. The addition of SeO_4_^2-^ or SeMet to the incubation medium was performed 50 s prior to the treatment with H_2_O_2_, after which fluorometric measurements were immediately started. CaCl_2_ (1 mM) was included (right panel) to assess the extent of Ca^2+^ entry. Data are expressed as means ± SEM from four independent tests. In both panels, at any given concentration of H_2_O_2_, statistically significant differences are indicated by different letters, whereas identical letters highlight non-significant trends.

The CaCl_2_, added to the incubation medium under these experimental conditions, did not increase the amount of Ca^2+^ entry compared to the control, despite the presence of H_2_O_2_ (**Figure 2**).

### Dose Response Curve of SeO_4_^2-^ or Se-Met in [Ca^2+^]_cp_ During Oxidative Stress Induced With 1 mM H_2_O_2_ in Control Pollen Grains

A dose response curve was performed *in vitro* by pre-incubating the control pollen grains marked with FURA-2AM and subjected to oxidative stress with 1.0 mM H_2_O_2_ with increasing concentrations of SeO_4_^2-^ or Se-Met (0–10 µM).

**Figure 3:**
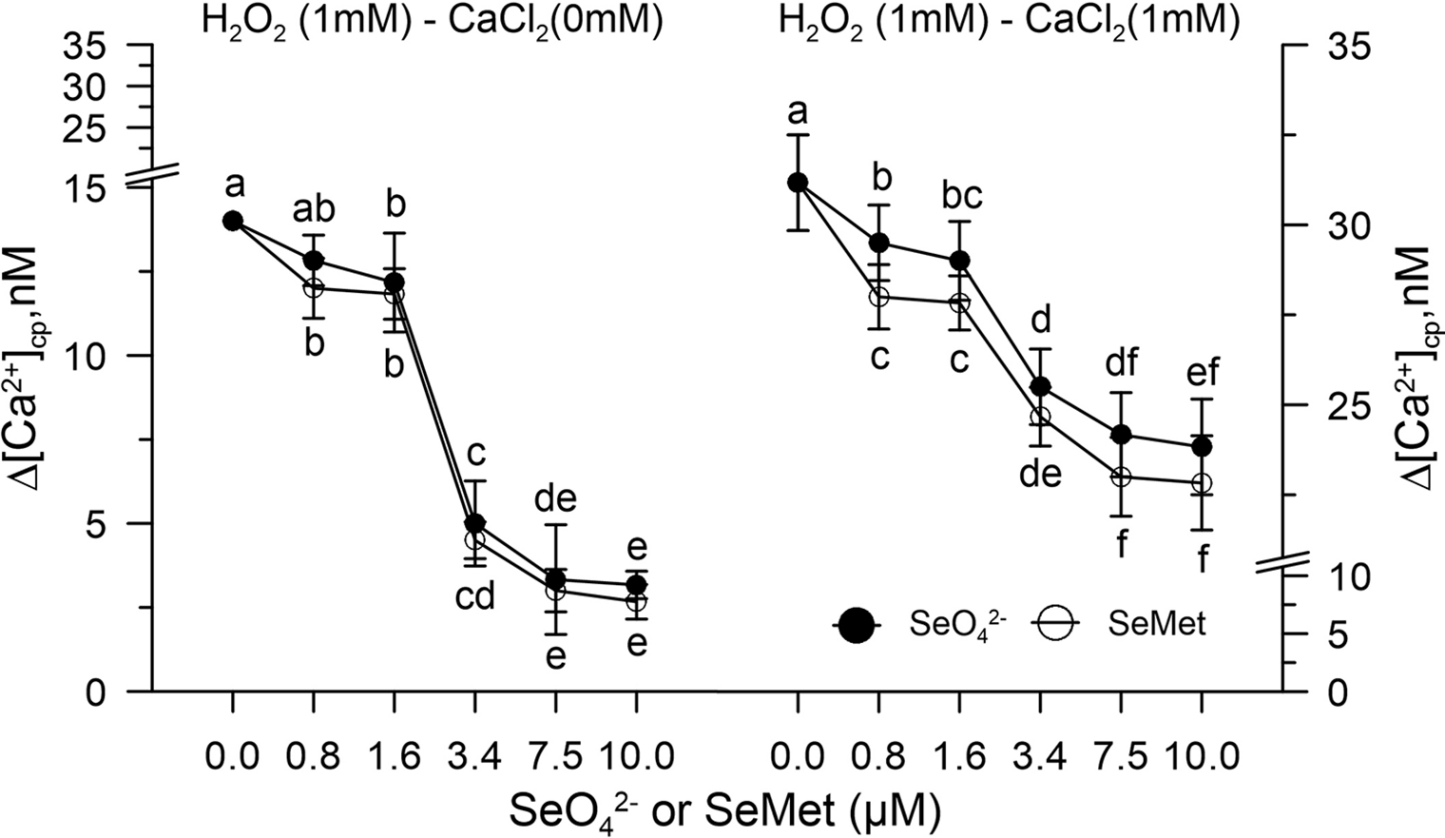
Dose response curve of SeO_4_^2-^ or SeMet (0–10 µM) in [Ca^2+^]_cp_ during oxidative stress induced with 1.0 mM H_2_O_2_ in control pollen grains, in the absence (left panel) and presence (right panel) of CaCl_2_ in the incubation medium. Data are expressed as means ± SEM from six independent tests. In both panels, at any given concentration of selenium, statistical significance of each set of data corresponding to a given dose of Se forms is indicated by different letters.

Hydrogen peroxide perturbed the homeostasis of [Ca^2+^]_cp_ by increasing its concentration in the absence of the ion in the incubation medium for release from the "internal stores." When the pollen suspension was pre-incubated with increasing concentrations of SeO_4_^2-^ or Se-met (0.8–10 µM), the effect of hydrogen peroxide on [Ca^2+^]_cp_ was inhibited starting from the concentration 3.4 µM of SeO_4_^2-^ or Se-Met (**Figure 3**), and the amount of Ca^2+^ entry after the addition of CaCl_2_ to the incubation medium did not increase with respect to the control in the presence of H_2_O_2_ (**Figure 3**).

### Effects of SeMet and SeO_4_^2-^ on Pollen Germination

**Figure 4:**
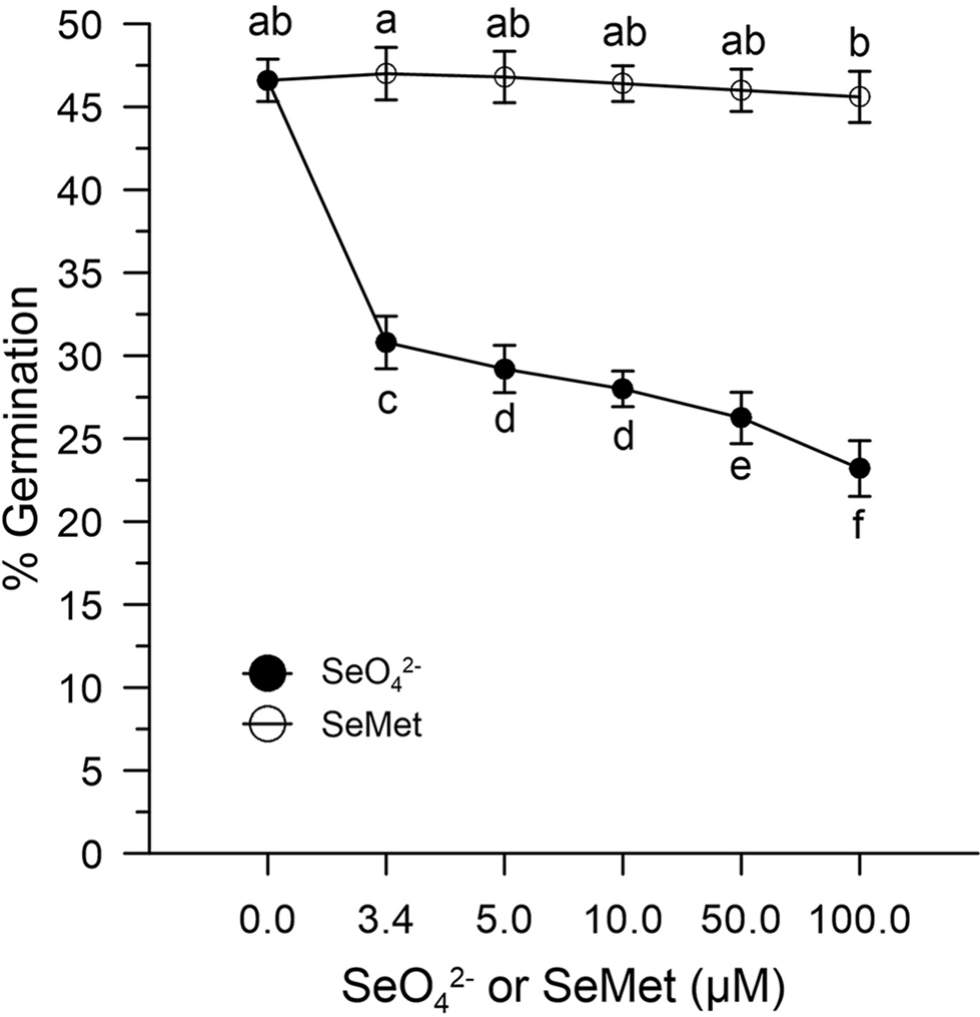
Effects of SeO_4_^2-^ and SeMet on germination rates. The graph shows data obtained from control olive pollen grains incubated with increasing concentrations (0–100 µM) of SeO_4_^2-^ (•) or SeMet (○). Results are expressed as % germination and represent the means ± SEM from five independent measurements, each of which supported by three technical replicates. Statistical significance of each set of data corresponding to a given dose of Se forms is indicated by different letters.

In the absence of oxidative stress, the germination capacity was determined by incubating pollen control samples *in vitro* with increasing concentrations (0–100 μM) of SeO_4_^2-^ or SeMet. **Figure 4** shows that the effect of SeO_4_^2-^was different from that obtained with SeMet. The pollen exposed *in vitro* to SeO_4_^2-^ showed rates reduced to about 30% compared to the 47% of the pollen exposed to the organic form (**Figure 4**).

### Impacts of Oxidative Stress Induced With H_2_O_2_ on the Germination of Control Olive Pollen Grains

**Figure 5:**
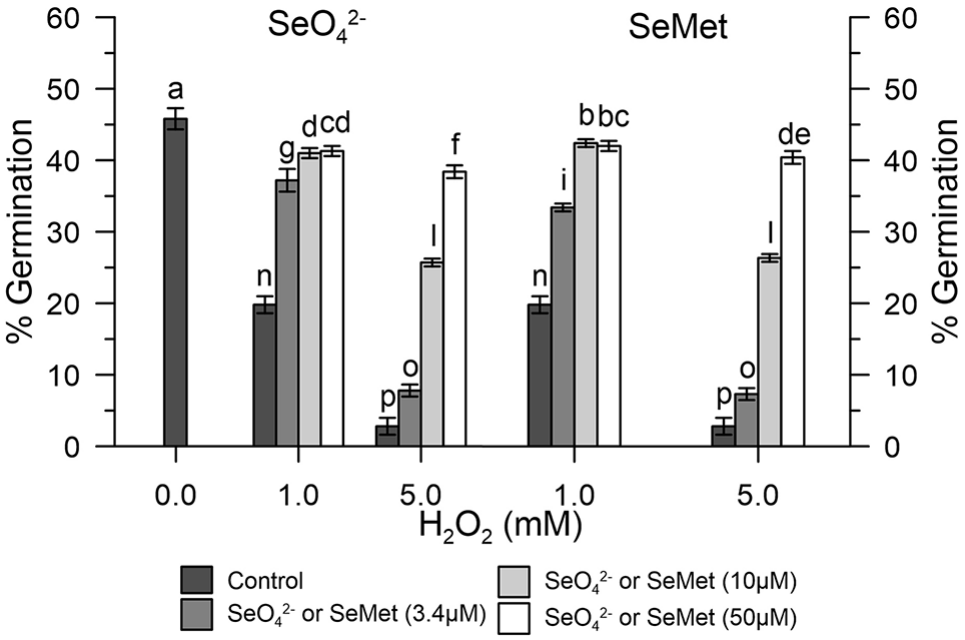
Germination rates under oxidative stress. The graph shows data obtained from control olive pollen grains pre-treated with increasing concentrations (0, 3.4, 10, and 50 µM) of SeO_4_^2-^ (left panel) or Se-Met (right panel), subjected to oxidative stress induced with H_2_O_2_ (0, 1, and 5 mM). Results are reported as % of germinated pollen and expressed as means ± SEM from five independent tests, each of which included three technical replicates. Statistical significance of the data set is indicated by different letters.

Germination rates of olive pollen under normal conditions were determined *in vitro* with 1 and 5 mM H_2_O_2_. Hydrogen peroxide showed an inhibitory effect, which progressively diverged as the concentration of H_2_O_2_ increased. In fact, at the concentration of 1 mM H_2_O_2_, germination was reduced to about 20%, while at 5 mM H_2_O_2_, it was reduced at about 2–3% compared to the control (**Figure 5**). Treatment with 3.4 μM SeO_4_^2-^ or SeMet together with 1 mM H_2_O_2_ resulted in a germination rate of about 35%, which represents about 76% of efficiency in relation to normal conditions. In contrast, treatment with 10 μM SeO_4_^2-^ or SeMet together with 5 mM H_2_O_2_ resulted in a germination rate of approximately 25%, which represents about 55% of efficiency in relation to normal conditions (**Figure 5**).

### Impacts of Oxidative Stress Induced With H_2_O_2_ on the Germination of Se-Enriched Olive Pollen Grains

**Figure 6:**
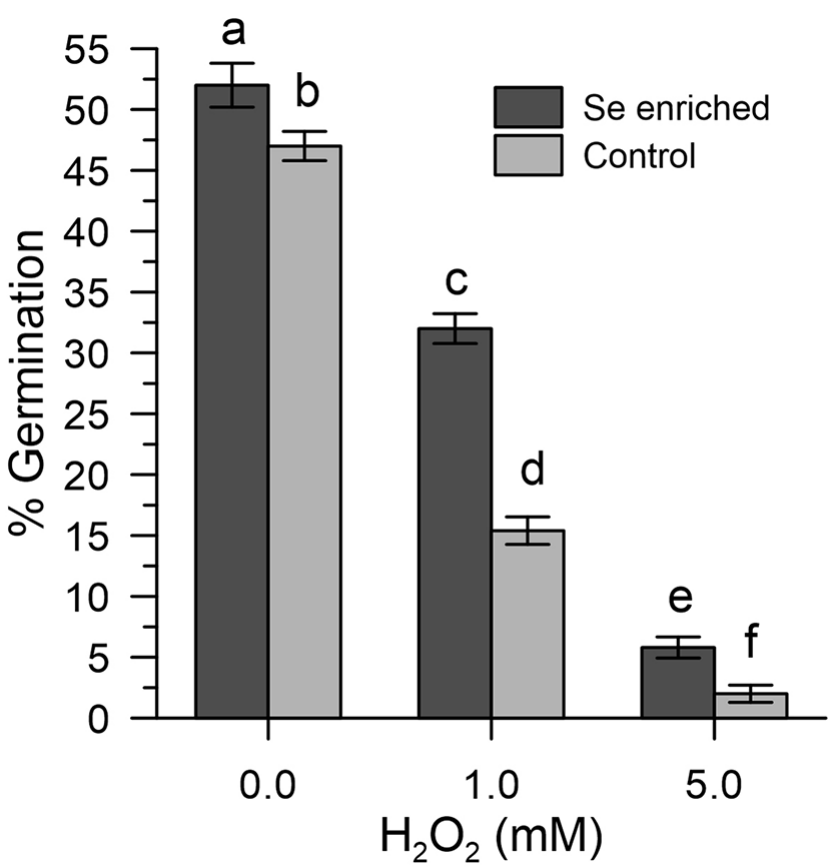
Percentage germination of Se-enriched and control olive pollen grains under oxidative stress induced with H_2_O_2_ (0, 1, and 5 mM). Data are expressed as means ± SEM from five independent tests, each of which included three technical replicates. Statistical significance of each set of data corresponding to a given dose of H_2_O_2_ is indicated by different letters.

The results obtained show that the germination efficiency of the two populations of pollen grains at basal conditions was different (**Figure 6**). The Se-enriched pollen grains showed a higher percentage germination than the control pollen grains (**Figure 6**). The 1 mM H_2_O_2_ reduced the germination of the Se-enriched pollen grains less significantly compared to the control pollen grains. The marked effects of 5 mM H_2_O_2_ on germination were negligibly removed in both populations of pollen grains.

## Discussion

In this paper, we show that pollen obtained from olive trees exposed to selenium fertilization has a superior capacity to counteract ROS-mediated effects that may negatively impact germination rates. The experimental steps consisted of spraying with sodium selenate, the collection of pollen from plants, and, subsequently, the application of H_2_O_2_ in pollen preparations to induce oxidative stress *in vitro*. Pollen samples, from plants treated or not treated with sodium selenate, were used to measure the cytosolic levels of Ca^2+^ and the pollen germination rates in the presence and absence of H_2_O_2_.

Cytosolic Ca^2+^ plays an important role as a secondary messenger, and alterations of Ca^2+^ homeostasis can induce molecular switches in the regulation and signaling networks of the pollen tube (Cheung and Wu, 2008; Steinhorst and Kudla, 2013; Görlach et al., 2015). Furthermore, the germinated pollen grains and the elongated pollen tubules require an internal Ca^2+^ gradient, which is maintained by an extracellular ion supply (Michard et al., 2009; Steinhorst and Kudla, 2013). Therefore, in light of the well-documented bidirectional relationship between ROS, which can modulate cellular calcium-dependent networks, and calcium signaling, which plays a key role in ROS assembly (Steinhorst and Kudla, 2013; Görlach et al., 2015; Proietti et al., 2018), we decided to use cytosolic Ca^2+^ as a final point for monitoring the perturbations caused by oxidative stress.

The results obtained show that H_2_O_2_-induced oxidative stress caused a significant alteration of Ca^2+^ homeostasis, evidenced by an increase in cytosolic Ca^2+^ and a markedly increased Ca^2+^ entry when exogenous 1 mM CaCl_2_ was added to the pollen suspension.

In contrast, Ca^2+^ homeostasis was fully maintained in control pollen exposed to sodium selenate or Se-methionine *in vitro* for 50 s before adding H_2_O_2_ and, immediately afterwards, at the start of measurements. Given the short incubation times, it is possible to rule out, at least *in vitro*, the implications of any metabolic mechanism and to conclude that Se, in both chemical forms, acts substantially as a simple scavenger of ROS, which eventually prevents ROS-mediated dysfunction of Ca^2+^ channels. Our results, however, indicate that the pollen obtained from the olive trees fertilized with Se, compared to the controls, consisted of about 40% of organic Se, thus suggesting that the inorganic form Se-enriched in the crops was metabolically and largely converted into Se-methionine (about 90%) and into other organic forms such as SeCys and MetSeCys.

The ability of Se-enriched pollen to tolerate oxidative stress (germination efficiency around 60% in Se-enriched pollen compared to 30% in the control with 1 mM H_2_O_2_) is particularly important for agricultural productivity, based on multiple abiotic factors that can lead to an excessive ROS production (Hasanuzzaman et al., 2013; Zinta et al., 2016; Fahd et al., 2017) and the observation made by others that show that abiotic stresses of different nature can inevitably lead to an excessive accumulation of ROS and, consequently, to pollen sterility (Lazzaro et al., 2005). Furthermore, it should be noted that Se-fertilization, despite having produced a total content of Se in the pollen about 11 times that of the untreated crops, did not affect the germination rates in the absence of oxidative stress. The high germination rates (about 50% vs. 46% in Se-enriched and control pollen) allow to exclude toxicity problems (**Figure 6**). However, as shown in **Figure 4**, inorganic selenium has a greater negative impact on the fertility of organic selenium, although a comparison between the data of Se-fertilization treatments and Se *in vitro* should be taken with caution, given the ability of fertilized plants to adapt to Se in the long term. In any case, any Se-fertilization protocol must be thoroughly validated by considering the multiple environmental factors, both biotic and abiotic, which can influence agricultural outcomes.

Probably, it is necessary to biochemically clarify the effects of Se on the pollen in order to optimize the conditions and to face possible unexpected events or, perhaps, to discover mechanistic events that could further improve the germination rates.

## Conclusions

In conclusion, we provide strong evidence, for the first time, of the beneficial effects of Se-fertilization in olive pollen. Furthermore, we demonstrate that the measurement of cytosolic Ca^2+^ is an easy and rapid determination that can be used to monitor the onset of oxidative stress and the efficacy of antioxidant measures. More years of investigations are necessary to better understand how selenium treatments can help the productivity of plants under stress conditions.

## Data Availability Statement

The datasets generated for this study are available on request to the corresponding author.

## Author Contributions

Conceptualization: PP, AP, CP, LR. Investigation: PP, LR, RD’A, AP, ET, DB, RD’A. Methodology: CP, AP, RD’A, ET. Supervision: PP, CP, DB. Writing—original draft: CP, AP, PP, LR.

## Funding

This work was supported by the EU project LIFE OLIVE4CLIMATE (LIFE15 CCM/IT/000141) and by the "Fondazione Cassa di Risparmio di Perugia" (Italy), Project: "Selenolivo", project code: 2015.0347.021.

## Conflict of Interest

The authors declare that the research was conducted in the absence of any commercial or financial relationships that could be construed as a potential conflict of interest.

## Supplementary Material

The Supplementary Material for this article can be found online at: https://www.frontiersin.org/articles/10.3389/fpls.2019.01611/full#supplementary-material.

Table S1 Dataset and statistical analysis for Fig. 1.

Table S2 Dataset and statistical analysis for Fig. 2.

Table S3 Dataset and statistical analysis for Fig. 3.

Table S4 Dataset and statistical analysis for Fig. 4.

Table S5 Dataset and statistical analysis for Fig. 5.

Table S6 Dataset and statistical analysis for Fig. 6.
